# Supplementary material for: Accounting for cell lineage and sex effects in the identification of cell-specific DNA methylation using a Bayesian model selection algorithm
Source: PLoS One. 2017 Sep 28;12(9):e0182455. doi: 10.1371/journal.pone.0182455 (PMC5619727; doi:10.1371/journal.pone.0182455)
Supplement: S1 Table — The category ‘Unassigned’ refers to all CpG probes that were not assigned to any marker panel, based on a 5% Bayes’ FDR. (PDF) [file pone.0182455.s001.pdf]

S1 Table: Percentage of markers associated with SNPs, for sex-specific and common markers by candidate model. The category 'Unassigned' refers to all CpG probes that were not assigned to any marker panel, based on a 5% Bayes' FDR.

Model	Female	Male	Common
$CD19^+$ B	24.19	26.82	27.05
$CD14^+$ Mono	37.02	34.62	36.51
$CD4^+$ T	29.05	28.34	26.10
$CD8^+$ T	23.14	19.47	21.97
$CD16^+$ Neu	37.11	33.85	38.84
$CD56^+$ NK	33.70	36.82	36.02
Pan T	24.43	24.79	25.65
Lymphocyte-I	28.03	27.55	28.46
Lymphocyte-II	30.68	27.88	30.91
Myeloid	34.22	30.58	34.92
All	34.84	34.62	35.46
Unassigned	26.81	26.75	26.61
