# Supplementary material for: Accounting for cell lineage and sex effects in the identification of cell-specific DNA methylation using a Bayesian model selection algorithm
Source: PLoS One. 2017 Sep 28;12(9):e0182455. doi: 10.1371/journal.pone.0182455 (PMC5619727; doi:10.1371/journal.pone.0182455)
Supplement: S2 Table — A sex-specific marker was declared if the posterior probability of a methylation difference ≥ 0.10 was greater than 0.95 for at least one cell type. (PDF) [file pone.0182455.s002.pdf]

S2 Table :Distribution of sex-specific common markers over chromosomes, for single cell-dependent makers only. A sex-specific marker was declared if the posterior probability of a methylation difference ≥ 0.10 was greater than 0.95 for at least one cell type.

CHR	$CD14^+$ Mono	$CD19^+$ B	$CD4^+$ T	$CD16^+$ Neu	$CD56^+$ NK
chr1	0	0	0	3	18
chr10	0	1	0	3	10
chr11	1	0	0	7	11
chr12	0	0	0	4	10
chr13	0	0	0	1	6
chr14	0	1	0	4	4
chr15	0	1	0	2	6
chr16	0	0	0	3	10
chr17	0	0	0	4	8
chr18	0	1	0	0	3
chr19	0	0	0	1	10
chr2	0	0	0	6	11
chr20	0	0	0	0	2
chr21	0	1	1	0	2
chr22	0	0	0	1	2
chr3	1	1	0	1	7
chr4	1	0	0	1	10
chr5	0	1	0	3	13
chr6	0	2	0	4	10
chr7	0	1	0	4	20
chr8	1	0	0	4	16
chr9	0	0	0	3	3
chrX	1	5	4	2	0
Unique Genes	5	11	1	47	133
