# Supplementary figures and images for: Accounting for cell lineage and sex effects in the identification of cell-specific DNA methylation using a Bayesian model selection algorithm
Source: PLoS One. 2017 Sep 28;12(9):e0182455. doi: 10.1371/journal.pone.0182455 (PMC5619727; doi:10.1371/journal.pone.0182455)

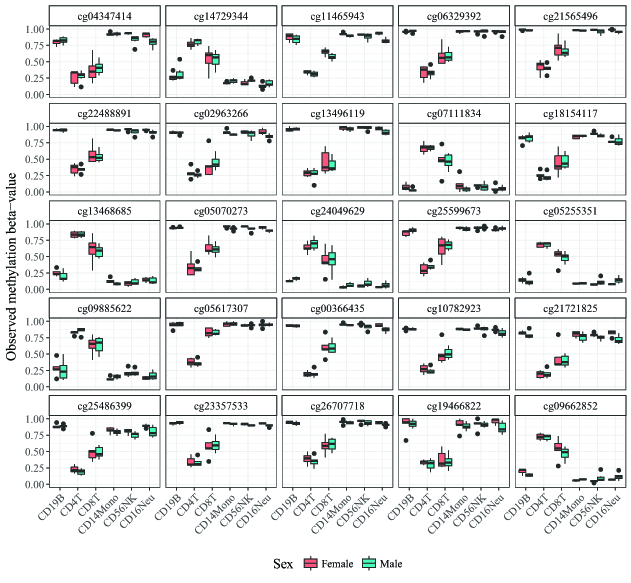

Supplement: S1 Fig — Markers were identified as having high levels of differential methylation (>0.5) in CD4+ T cells. Markers were identified if the corresponding posterior probability of differential methylation >0.5 exceeded 0.95. (TIFF) [file pone.0182455.s003.tiff]

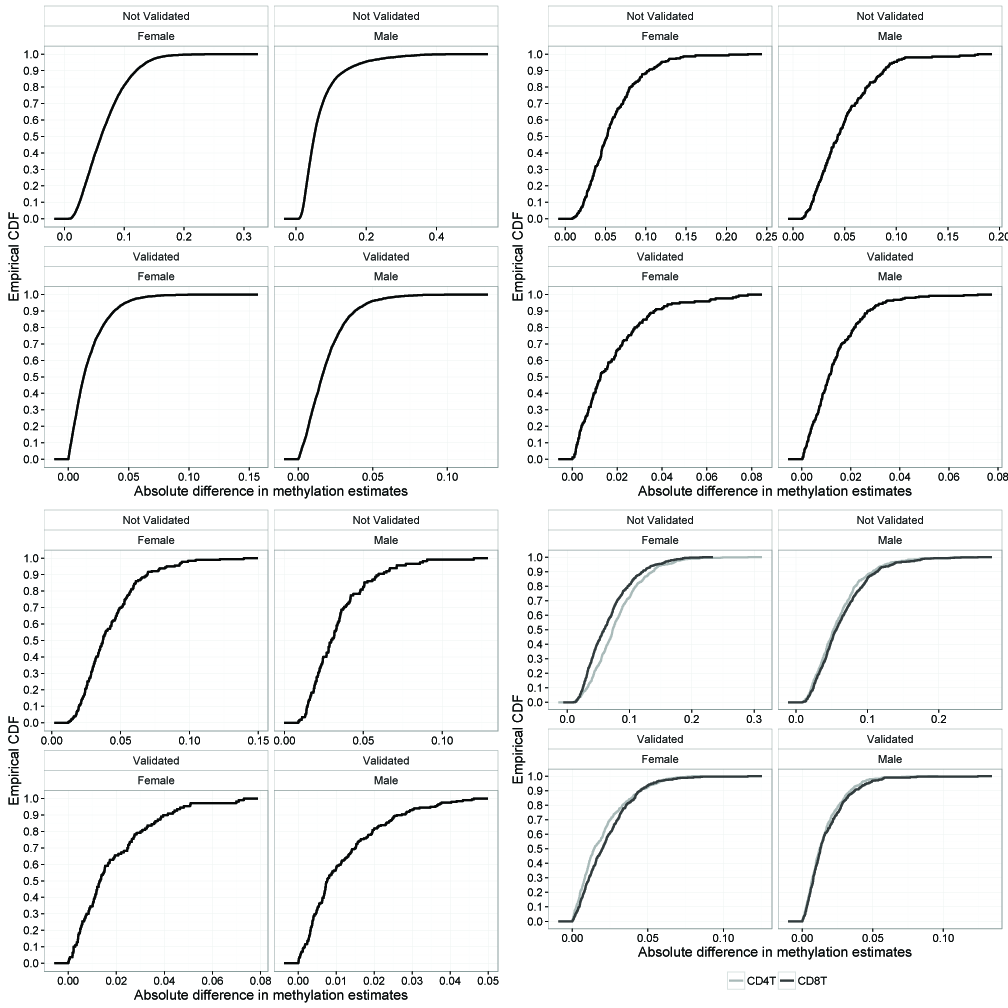

Supplement: S2 Fig — Validation status was defined by the coverage of each 95% CI, estimated from the training data. First row (L-R): CD19+B, CD4+T; Second row (L-R): CD8+T, Pan T. (TIFF) [file pone.0182455.s004.tiff]
